# Why ignore expiry dates on cosmetics? A qualitative study of perceived risk and its implications for cosmetics producers and regulators

**DOI:** 10.1111/risa.70040

**Published:** 2025-04-21

**Authors:** Yujiao Wang, Gary Davies, James Derbyshire, Farid Ullah

**Affiliations:** ^1^ The Business School Edinburgh Napier University Edinburgh UK; ^2^ Alliance Manchester Business School University of Manchester Manchester UK; ^3^ Chester Business School University of Chester Chester UK

**Keywords:** cosmetics, expiry date, perceived risk theory, public health, public policy

## Abstract

Consumers often use cosmetics long after their expiry date, despite the health risk. This paper aims to understand why and to suggest policy changes that can promote safer practices in cosmetics use. This is the first study to investigate risk perception in relation to expired cosmetics. Thirty‐three semistructured interviews with both cosmetics users and employees of cosmetics companies were conducted in the United Kingdom and China. Perceived risk theory was found to be a useful analytical lens. Eight risk factors emerged from the data, including two not previously identified. Combinations of risk were also found to be valuable in explaining consumer attitudes to cosmetic expiry dates, which suggests that perceived risk factors interact with each other to create an emergent perception of risk, requiring an integrated understanding. While physical, performance and self‐brand connection risk can promote adherence to an expiry date, other risk factors such as financial and social risk can override such concerns, leading to the expiry date being ignored. Implications for suppliers’ and regulators’ policies and risk‐communication strategies are identified that may help reduce the risks being taken by cosmetics users.

## 1 INTRODUCTION

Using cosmetics beyond their expiry date can represent a significant health risk, as the quality and safety of many such products deteriorate over time, particularly after they are opened (FDA, 2022). There are two types of cosmetic expiry date: its shelf life if unopened and its Period After Opening, or PAO, which is its shelf life once opened. This paper focuses on PAO because, while unopened cosmetics can deteriorate, chemical changes in and/or contamination of cosmetics generally occur after opening (Skowron et al., 2017).

There are no global standards governing expiry date labels on cosmetics, but similar approaches exist worldwide. In Europe, cosmetics with a lifespan over 30 months must have a PAO date (Cosmetics Europe, 2023). China mandates either a production date and shelf life, or a batch code and PAO date on packaging (Dorato, 2018). In the United States, expiry dates are not mandatory, but suppliers must ensure safety (FDA, 2022). In practice, most companies use an opened jar symbol with a number and a capital M to denote the PAO in months. Its use was, for example, mandated in Europe in 2009 to update existing guidance (Cosmetics Europe, 2011) and to improve the protection of human health. Expired cosmetics can harbor high levels of microbiological contamination that pose a health hazard to users (Giacomel et al., 2013).

There is evidence of consumers suffering adverse reactions (e.g., allergic reaction and infection) and skin problems from continuing to use out‐of‐date cosmetics, including acne, irritation, and skin rashes (Giacomel et al., 2013; Šniepienė & Jonuševičienė, 2019). While this is somewhat ironic because the purpose of using cosmetics is to improve appearance (Mafra et al., 2020), the potential consequences from the use of expired cosmetics are not merely aesthetic and can be serious, including eye infections, urinary and respiratory tract infections, and even meningitis (Al‐Rifaaia et al., 2021; Ng et al., 2016). There are several causal mechanisms by which an expired cosmetic might deteriorate leading to negative health effects, such as microbiological contamination from exposure to the environment, or its user transferring bacteria or mold to a cosmetic (Giacomel et al., 2013). Contamination becomes more likely the longer a cosmetic product is used. From analyzing large samples of used cosmetics, prior work has found high contamination rates of between 70% and 90%, and that expired cosmetics had the highest microbiological contamination (Bashir & Lambert, 2020; Skowron et al., 2017). However, and despite use‐by labeling having been widely adopted, Giacomel et al. (2013) found that more than 90% of women use cosmetics beyond their PAO, including mascara, eye pencil, lipstick, and eye shadow, which are among the types of cosmetic most often linked to the risk of infection.

One explanation for why consumers continue to use expired cosmetics is a lack of awareness of the risk of infection (Özdemir et al., 2019); another is that there is no simple way for consumers to monitor their cosmetics expiration other than by remembering/recording the date of purchase and first use, and following informed advice available to them (e.g., online from medical practitioners (Logan, 2021)) such as watching for signs of deterioration, including changes in color, texture, or odor. Expiry dates can vary by product, ranging from as little as 3 months after opening for mascara to up to 2 years for lipstick and between different brands of the same type of product, making such monitoring difficult. However, there is a paucity of empirical evidence supporting these explanations for consumers’ disregard of cosmetic expiry dates, or on which to base alternative explanations. There is therefore a need for more research on why consumers do not adhere to PAO labeling and do not dispose of cosmetics on time. This can assist policymakers, manufacturers, and retailers in designing policies that respond to the problem by effectively communicating the risks to consumers and addressing this important knowledge gap.

We report findings from semistructured interviews with cosmetics consumers and those working for cosmetics suppliers, which we analyze through the lens of perceived risk theory. The findings illustrate how consumers trade off the risk of adverse health effects from ignoring PAO information against other, non‐health‐related, perceived risks, including psychological, financial, and social risk, to justify not following manufacturers’ guidelines. In analyzing our data, we identify two risk types not reported in prior work, and uncover important interactions between risk types, which have not been made explicit previously.

In what follows, there is first a review and definition of perceived risk and a description of the sample of respondents. That is followed by a description of the methods employed in the study, and how the analyses of the transcribed interviews and interviewer notes evolved. This leads into Section 3 where the usefulness of perceived risk theory as a theoretical lens for analyzing the reasons for consumers ignoring cosmetic expiry dates is illustrated. A discussion of the findings follows, in which we develop new theory and make recommendations for regulation and policymaking that can mitigate the risks associated with the use of expired cosmetics. Finally, the limitations of the present study and its implications for future research are discussed.

### 1.1 Perceived risk

Perceived risk can be understood from a consumer perspective as the product of the subjective probability of the (risky) outcome occurring and the negative consequences if it does (Mitchell, 1992). For example, the greater the perceived risk of a threat to health, the more likely the individual is to act to counter the actual threat (Rundmo, 1999). Perceived risk is then a measure that combines the expected dissatisfaction associated with the outcome of an action and the probability of that outcome occurring (Pires et al., 2004). It represents a subjective means of assessing potential gains and losses, as customers evaluate risk according to their individual perspectives and experiences (Slovic et al., 2004).

While some prior work on consumer risk treats it as a summary construct (Dowling, 1986; Li et al., 2020), other work (e.g., Mitchell, 1992; Zanetta et al., 2022) has argued that perceived risk is multidimensional. This suggests it is useful to consider its component parts. Prior work (e.g., Mitchell, 1992; Tsiros & Heilman, 2005) identifies six dimensions of perceived risk: social, financial, psychological, performance, physical, and time (defined later in Section 3). Stone and Grønhaug (1993) have illustrated how each can contribute to overall perceived risk and how they can interact in doing so.

Research linking consumers’ understanding of cosmetic expiration dates and perceived risk is scarce. While some researchers mention the potential link between expiry date and purchase/repurchase intention (e.g., Thakar & Patel, 2013), none have researched the link explicitly. Some research has considered the risk from cosmetics from a consumer safety perspective—for example, the possibility that talcum powder might contain asbestos (Burns et al., 2019; Holton et al., 2022), which is somewhat distinct as an issue from that of the expiry date that is our focus here.

Logically, the higher the perception of a risk—for example, the risk of damage to health from the use of expired cosmetics—the more likely it is that there will be behavior designed to counter it, such as, in this case, the disposal of an out‐of‐date cosmetic product. However, and just as perceived risk theory emphasizes, there may be several negative consequences associated with disposal, which might offset the impulse to dispose of a cosmetic in a timely fashion and avoid the risk of adverse health consequences.

## 2 PARTICIPANTS AND METHODS

### 2.1 Sample

As women dominate the consumption and sale of cosmetics (Allied Market Research, 2023), in the qualitative research that follows, participants were either female users of cosmetics, or they were female employees of cosmetics companies. In total, 33 women (19 from China and 14 from the United Kingdom) participated in the research, 10 as employees and 23 as consumers.

Two factors were used to select the sample of consumers: their age and nationality. The rationale is that consumers’ attitudes toward cosmetics and their use change with age (Ramshida & Manikandan, 2014), and attitudes can also differ between cultures, such as between Eastern and Western cultures (Madan et al., 2018). Cross‐cultural sampling can increase the validity of research findings (Tsui et al., 2007). The two countries selected, China and the United Kingdom, are both major markets for cosmetics (Statista, 2022) and differ in culture^1^ (CFG, 2024). It is important to explore whether the perceived risk theory effectively explains both the UK and China data. In both markets, interviews were also made with employees of cosmetics companies.

### 2.2 Method

A bilingual researcher undertook all interviews in 2021 and 2022. They took place either online (14) or in person (19). Pilot interviews were used to pretest the interview guide and questions. As there was the potential for consumer responses to be affected by social‐desirability bias, a projective technique was adopted (Krumpal, 2013). Photographs were downloaded from the Internet of women's faces in full makeup representing different age groups and both nationalities. Consumer respondents were asked questions about what they imagined the woman in the picture would do or think if she believed a cosmetic product she was using was beyond its PAO expiry date. In the process of talking about other people, interviewees would reveal their own attitudes, and, when these were out in the open, they would start to discuss these more freely (Rojas‐Méndez & Davies, 2024). Consumers would then be happy to discuss the cosmetics in their possession and, frequently, why they had not adhered to use‐by dating.

As little previous research had been done around the topic of cosmetics expiration dates and consumer behavior, the initial approach to data analysis adopted the Straussian version of grounded theory (Strauss & Corbin, 1997). As is typical in grounded theory work, theoretical sampling was adopted and the selection of respondents evolved as the work progressed. Initial consumer respondents were identified through the researchers’ social networks and were then asked to recommend other potential contacts who could meet the sampling criteria (Parker et al., 2019). Early interviews with customers included mention of the advice from in‐store salespeople on cosmetics disposal. Consequently, eight employees of cosmetics companies were recruited by approaching them in‐store to benefit from their insights into why customers might not dispose of out‐of‐date products. Partway through data analysis, which took place concurrently with the interviews, it became clear that many of the issues raised by interviewees concerned the policies of suppliers—both retailers and manufacturers. Consequently, additional interviews with a buying manager for a cosmetics retailer and a director of a cosmetics manufacturer were obtained. With the consent of the respondents, most interviews were recorded. Two interviews with industry representatives were not recorded as they took place in‐store. Here, the researcher took notes during the interview and confirmed the key points with the participants immediately after the interview (Bleich & Pekkanen, 2013). For the other interviews, the researcher also took notes of key points and sometimes observations of nonverbal communication to help clarify the meaning of what was said (Middendorf & Macan, 2002).

Because peer‐group influences can be strong in cosmetics use, as evident in the sharing of products (Sim & Lee, 2021), two group interviews with consumers were conducted—one with two and another with three respondents at the same time. In each group, the participants knew each other well. We therefore carried out both individual and group interviews with consumers, and interviews with employees of cosmetics companies, to increase the richness and trustworthiness of our data (Lambert & Loiselle, 2008). Interviews lasted between 20 min and 2 h. They were transcribed verbatim and translated into English from Chinese as appropriate.

Initially we were heavily influenced by grounded theory (Strauss & Corbin, 1997), and no prior assumptions were made about any existing theory that might explain our data or guide its analysis. However, from initial analyses of the interviews, it became clear that perceived risk theory could be used to structure much of the analysis, with the theory's component risks representing individual themes. Thus, thematic analysis was adopted with perceived risk theory's component risks used to guide the recoding of all interviews (Braun & Clarke, 2006). However, the process of data coding also allowed the development of themes beyond these preestablished codes, amounting to wholly new risks.

The interview transcripts and field notes were analyzed and coded using NVivo 12. The lead researcher conducted the main coding of the data, while a second researcher checked her examples with the transcripts and field notes (Berger et al., 2023; Braun & Clarke, 2021). The interpretation of quotes that appeared to represent more than one type of perceived risk and the definition of two new types of perceived risk that emerged from the analysis were discussed in depth. Our approach to thematic analysis therefore combined Braun and Clarke's (2019) reflexive approach, which is an iterative process of constantly questioning and querying the assumptions made in interpreting and coding the data, with template analysis (Byrne, 2022). The template analysis was based on the framework provided by perceived risk theory. In total, eight component types of risk were identified, each relevant to understanding the respondents’ overall perception of the risk from using out‐of‐date cosmetics.

## 3 RESULTS

A selection of quotations illustrating each type of risk is discussed in the following sections, together with relevant prior work to help substantiate each. Individual quotes are coded (e.g., FR1 refers to the first quote in Section 3.1). A second code denotes the respondent (e.g., CC1_20‐29 indicates Chinese Consumer participant 1 aged between 20 and 29, and UKC1_20‐29 for UK Consumer participant 1 aged 20–29; while EC1 denotes Employee 1 working in the cosmetics industry in China, and EUK1 refers to Employee 1 in the United Kingdom).

### 3.1 Financial risk (FR)

Cosmetics can be an expensive purchase that leaves consumers with a sense of financial loss when disposing of them (quote FR1), representing a financial risk, which is defined as: the product will not yield the expected economic benefit to the consumer (Mitchell, 1992). The idea of loss aversion is relevant here (Morewedge & Giblin, 2015), although affluence also appeared likely to override any concerns over financial loss (FR2).

*FR1: “Especially expensive cosmetics, I'm reluctant to throw away.” (CC10_20‐29)*
*FR2: “I don't think she will use expired cosmetics. She's a working woman and looks rich.” (*referring to a photograph of a young woman wearing full makeup*) (CC9_20‐29)*
*FR3: “I was going to bin them, but my mom saw them and she thought it was too wasteful to throw them away, so she used them. She said it would be fine for her face. But I won't use them.” (CC13_20‐29)*

Views could differ between respondents on the same disposal issue (FR3). One respondent had bulk‐purchased facial masks but had not used them before they expired. She had wanted to throw them away, however, her mother thought this was wasteful and used them herself. This might suggest that age can influence cosmetics disposal, with older people being more conservative and less inclined to discard out‐of‐date cosmetics. Prior work (Johnson et al., 2006) also indicates that older individuals tend to be more loss‐averse, and we return to the possible influence of age on disposal later. Loss aversion is also useful to explain why people might avoid the disposal of expired expensive cosmetics or large stockpiles of old cosmetics—that is, because they are more sensitive to financial losses (Wilson et al., 2008) than to gains (Kleinübing Godoi et al., 2005).

### 3.2 Physical risk (PHR)

Although not all consumers recognized the PAO symbol, all acknowledged a possible PHR from using out‐of‐date cosmetics, defined here as: use of the product will jeopardize the health of the consumer. Those with an awareness of potential skin damage cited fear of its consequences when explaining their decision to dispose (PHR1 and PHR2).

*PHR1: “I throw expired products away. At my age, it will take a long time for my skin to recover from harm, like acne, inflamed skin. I don't want to take that risk.” (CC4_40+)*
*PHR2: “It's mascara that gets me, because it's so close to the eye. If you put something (eye shadow) on the lid, you've got the eyelash between your eye and the cosmetic. I don't take any risks with mascara at all.” (UKC1_40+)*
*PHR3: “But I feel if it's an eyebrow pencil or mascara, which is applied on a small part of your face, it doesn't matter.” (CC8_20‐29)*

Two different opinions are highlighted, with an older consumer saying she would take no risks with mascara (PHR2), while a younger consumer argued that because its use was on such a small part of her face, she could ignore the risks (PHR3). Another implied that younger people might be more willing to take risks with cosmetics because their skin could easily recover (PHR4). In reality, out‐of‐date mascara, due to its high water content, represents a significant microbial contamination risk to health just 3 months after opening (FDA, 2022). Rationalizing risk (PHR3 and PHR4) is a way of coping with the cognitive dissonance caused by a conflict between behavior and knowledge of that risk (Cao & Just, 2010).

*PHR4: “Just like we think we're young, it doesn't matter”. (CC9_20‐29)*

### 3.3 Performance risk (PER)

If a cosmetic deteriorates, its performance can decline. PER (defined as: the product will not work well or in a way that satisfies the consumer) can be the easiest risk for users to acknowledge, as it is often manifest in an obvious physical change to the product. For example, mascara might dry up or liquid foundation might separate, suggesting the product will no longer function as expected. When considering a purchase, PAO should be a predictor of how long a product will maintain its functionality. If a product does not fulfil its promise, then it will not satisfy the consumer (Mitchell, 1992). In the interviews, PER perceptions appeared closely related to the purchase process (quotes PER1, PER2, and PER3) (see also Mitchell, 1992). One complained that PAO information was often unavailable when buying online (PER1). Online customers will generally only find out about cosmetics’ PAO after paying for and receiving their purchases. Cox (1967) argued that there is a predictive value in such a piece of information—a “cue” regarding the future performance of the product. Expiry‐date information acts as a cue without which online consumers are at a disadvantage because they will be unaware of how long a product might last until they read the PAO label after the product arrives.

Where there is an obvious change to the physical state of the product after expiration, such as a change to its smell or texture (PER2), a consumer may decide to get rid of it. But they could also ignore the PAO and timely disposal when PER is seen in the context of another perceived risk, such as FR (PER3). The perception of a financial loss may be weighed against PER, reducing or even overriding the impulse toward disposal from the perception of PER. This suggests that individual risk factors are not independent of each other, which is something implied in Stone and Grønhaug (1993) that we return to later in Section 3.9.

*PER1: “Some of the cosmetic product details pages will show expiry date information, but some don't.” (CC13_20‐29)*
*PER2: “If the smell or the texture was bad, I'd get rid of it.” (UKC5_30‐39)*
*PER3: “If I can see the date…um I will look at it but if it had six‐month and it wasn't for everyday use, I'd really have to think about it, because I don't know a thousand percent would I wear it? like wait I'd be very conscious of its performance. I wouldn't bin it after six‐month if it's expensive. If it says ideally 12–36 months, I'd be like okay that's worthwhile.” (UKC7_20‐29)*

### 3.4 Psychological risk (PSR)

A PSR (defined here as: when the product or its use might negatively affect the peace of mind or self‐perception of the consumer) can threaten a customer's self‐image. Referring to a photograph of a mature woman, one respondent implied that the woman would not risk her self‐image by using out‐of‐date cosmetics (PSR1). Another, referring to her own experiences, said she would feel “uncomfortable” using an out‐of‐date product and would replace it (PSR2). She repeated the word “uncomfortable,” emphasizing her point. Both comments illustrate potential damage to self‐esteem, with one coping mechanism being to dispose of the cosmetic.

*PSR1: “This lady is my mom's age. She looks very gentle and elegant. She probably wouldn't use out‐of‐date makeup.” (CC9_20‐29)*
*PSR2: “According to the PAO, it has expired. I feel uncomfortable to use it and will replace it with a new one. Although I don't know what effect it will have on my skin after the date, I feel uncomfortable.” (CC3_20‐29)*

The tendency to strive to feel good about oneself is a fundamental aspect of human nature, but it is also linked to how individuals feel they are evaluated by others (Leary, 1999), which is discussed next.

### 3.5 Social risk (SR)

SR—that is, whether one's behavior will negatively affect other individuals’ perceptions of one—concerns the risk to how a person is seen by others. Here, that would involve their relaxed attitude toward expired cosmetics becoming known and creating a negative impression. SR was evident in one respondent's concern about how she would be seen by her friends (SR1). She not only cared about her friends’ advice for expired cosmetics but also how they would view her trading off between the potential health issues caused by expired cosmetics and the cost of repurchasing them. Note that she implied she would only be willing to show her “funny” expensive products to her friends, and not the cheaper ones, implying an image consciousness that can be associated with SR.

*SR1: “I might try to investigate a bit more with a more expensive product, like funny I'm gonna show everyone these do you think this is not funny? but if it's a cheap product, I will probably be like, oh, this is not funny I'm gonna throw it away, straight away. I'm not going to ask anyone else because obviously if it's a cheap product, and it's like you're not losing a lot.” (UKC2_30‐39)*

Cosmetics can be fashion items (e.g., the latest and most fashionable color of lipstick). Here the social issue is to be seen as fashionable, which may be another factor that offsets or overrides any risk to one's health from the use of an expired product. Two similar views from salespeople (SR2 and SR3) corroborate this, implying that lipstick would be purchased because it is fashionable, and would be disposed of for the same reason (i.e., because it is no longer fashionable), rather than because it has an expired PAO. Younger consumers appear to be more influenced by SR in that they are particularly sensitive to what their peers think of them, which reflects similar findings about clothing (Maziriri & Mokoena, 2016).

*SR2: “Usually we buy it, just like young people, generally don't care too much about this (shelf‐life), really, only care about whether it looks good or not.” (EC1)*
*SR3: “Young consumers don't. Young customers read advertisements/posts. After the new lipstick comes out, they will immediately buy a new one. They won't care about PAO, no.” (EC2)*

### 3.6 Time risk (TR)

Relatively few examples of time‐related risk emerged from the interviews. Where this risk was identified, it interacted with one of the other risk types (we discuss the interaction between risk types in more detail in Section 3.9). Both examples of TR (TR1 and TR2) involved interaction with PSR from negative shopping experiences and concerned the time and effort involved in returning cosmetics purchased online, reflecting the general literature on risk when shopping online (Featherman & Pavlou, 2003). No examples emerged of TR influencing disposal.

*TR1: “We're very passive on online shopping, we cannot see the production date or expiry date, most of the time you receive it and accept it. I may not buy things from this online seller again even though I quite liked them before. Only if it's really out‐of‐date for a long time, I will ask for an exchange otherwise it's too time consuming.” (CC12_20‐29)*.
*TR2: “I bought a XXXX's cream on their website sales, it was about £50, however I bought it in 11/2018, the expiry date is in 01/2019. So I took the extra time to contact customer service and they refunded me half of the price. It was an unpleasant shopping experience.” (An online review mentioned by respondent UKC9_20‐29)*

TR is apparent if a purchase could make the consumer lose convenience, or waste time/effort, for example, by seeking a product replacement, a refund or a return (TR1 and TR2). As mentioned above, when purchasing cosmetics online, the expiry date is often only known to the consumer after delivery. In TR1 and TR2, consumers reported negative experiences when online purchases arrived near the cosmetic's expiration date, leading to a PSR and affecting repurchase intention.

In addition to the six risk types from the existing perceived risk framework, two new risks emerged from the analysis, labeled here as Self‐brand connection risk (SBCR) and Environmental risk (ENR).

### 3.7 Self‐brand connection risk (SBCR)

Cosmetics companies may invest heavily in relationship building with customers (Hodge et al., 2015). Consumers can be very loyal to their favorite cosmetics brands, as evidenced by one interviewee (UKC1_40+) who decided to travel 60 km to another city to continue receiving personal advice from her preferred brand after her local department store had closed.

In the context of a PAO, providing full expiry information can reassure a customer (SBCR1) and influence brand imagery (SBCR2). This can help in building a strong relationship between a brand and a consumer. If a brand reduces risk, it is more trusted (Siegrist, 2021). Helping consumers keep track of the expiration of their purchased cosmetics could benefit a brand and encourage timely disposal (SBCR3).

*SBCR1: “Complete date information will make me feel safe and confident to buy from this brand. It will make me think something bought from a prestigious brand is always better value.” (CC3_20‐29)*
*SBCR2: “A product with (a clear) PAO is slightly more formal and reliable.” (CC9_20‐29)*
*SBCR3: “I would be quite happy with a tool to notify me the about date information and when to dispose a product. I would buy again if I had a good experience with the brand and if I liked it.” (UKC4_20‐29)*

There were examples of PAO‐related matters having both a positive and a negative effect on relationship building across the interviews. For example, two respondents interviewed together discussed purchasing the same eyeliner (SBCR4 and SBCR5). One had not used the purchased cosmetic before its relatively short expiry date, by which time the product had deteriorated, while her friend avoided this problem because she had received and followed the advice from a sales assistant who made her aware of the product's short shelf life. The former blamed her negative outcome on the lack of advice from the sales assistant, while the latter felt a stronger connection to the brand due to having received useful guidance. This meant one identified with the brand and the other not. Consumers value relationships with brands which help define and project their identity (Kressmann et al., 2006).

*SBCR4: “I bought a XXXX eyeliner. It was good looking, with a frosted bottle, but after I bought it, I completely forgot to use it regularly. When I took it out again, it was no longer usable. The liquid had dried out. The sales assistant didn't mention expiry date or when I should dispose of it.” (CC11_20‐29)*
*SBCR5: “But I've bought XXXX's liquid eyeliner several times, I bought it at YYYY just like you. If the sales assistant is a local, she would tell me that I should pay attention to this short expiry date.” (CC10_20‐29)*

Consequently, SBCR is defined here as: the perceived negative consequences on the relationship formed between consumers and brands, and the risk of losing that connection for the consumer. To reduce SBCR, consumers could be better informed by brands, encouraging them to follow expiration instructions and leading to disposal. SBCR is therefore not merely a business‐ or marketing‐related risk that applies only to the supplier or producer of a cosmetic, having no bearing on the individual consumer and her health. There is a clear connection between SBCR and risk to consumer health. The evidence we have presented above suggests that the development of a connection between a brand and a consumer may assist in ensuring that the consumer is cognizant of and follows guidance regarding expiration dates.

### 3.8 Environmental risk (ENR)

We define ENR as the consumer perceiving there to be a risk to the environment from throwing away unused cosmetics. This risk emerged mainly from the interviews with UK respondents, a finding compatible with De Silva et al. (2021), who suggest such concerns are more likely in the United Kingdom than in China—something that was further confirmed by the interviews with two senior managers (EUK3 & EUK4).

Perception of this risk means consumers feel better using up an expired cosmetic rather than disposing of it because they are worried about how any leftover product will be disposed of and the waste of resources associated with doing so. Three quotations (ENR1, ENR2, and ENR3) illustrate the point and are suggestive of a real enthusiasm for cosmetics with environmentally friendly credentials, such as those with recyclable containers, even if they have higher prices.

*ENR1: “Something I would like to see more is containers where they take empty bottles or empty lipsticks…that puts me in the mode of buying those brands because I want to look after the planet. It makes me feel better.” (UKC2_40+)*
*ENR2: “I think people that are a bit more environmentally friendly are the ones that would want to, not throw away but find a way to deal with it, yeah I'm environmentally friendly too” (UKC3_20‐29)*
*ENR3: “If you look at XXXX as an example, it's a relatively entry price point brand but it's all about recycling, sustainability, clean ingredients, vegan etc. So you can do it, it doesn't have to be about price. We are now in a generation because of global warming and all the other things, this is a real energy about you know we need to be cleaner, we need to be better.” (UKC10_30‐39)*.

### 3.9 Interaction of risk perception factors

As illustrated earlier, some issues could relate to more than one risk factor simultaneously, which is something that prior work has not discussed sufficiently. In other words, risk factors are not necessarily independent of each other, requiring an integrated understanding. For instance, the following single quote links four risk factors:

*“For powder cosmetics whether I throw away depends on their texture. I regularly throw away expired ones. Expensive ones, I may not throw away, but normally I do. But for expired blusher or eye shadow, if I really like it, I still keep it. Cause it's applied after the foundation layer, I feel it's fine for my skin.” (CC13_20‐29)*

FR is acknowledged here in terms of being less likely to throw away a more expensive item. PHR is also acknowledged in the (incorrect) perception of there being less PHR associated with using expired blusher or eye shadow than from other cosmetic types because both are applied over a foundation layer. PER is also acknowledged in the implication that disposal of cosmetics depends on their texture (e.g., powder or liquid), thus implying a link to the functionality of the product. Finally, failing to dispose of an expired product because it is liked and the sense of loss from the prospect of disposing of a valued item are suggestive of a PSR.

## 4 DISCUSSION AND CONCLUSIONS

The main aim of this paper is to provide a better understanding of the risk factors associated with consumers ignoring or complying with PAO guidance on cosmetics. The length of a PAO expiry date is determined by considering the physical, chemical, and microbial state of the product and how this will change over time. Consequently, one might expect PER and PHR to be foremost in the minds of consumers when considering a cosmetic's PAO and its subsequent disposal. However, our interview evidence shows that consumers may trade off these risks and other perceived risks. FR emerges as a main cause of continued use beyond the expiry date, especially if the product is seen as expensive. ENR can also delay UK consumers’ disposal of a cosmetic if disposal involves environmental concerns. These risks may offset or even entirely override the impulse to dispose of a cosmetic that stems from any perceived PER or PHR.

In addition, certain risk types can have both positive and negative effects on adherence to a PAO expiry date. A consumer may be less or more likely to dispose of cosmetics in a timely manner depending upon their perception of SR and PSR. Consumers can ignore a PAO if the product is seen as fashionable, or if their peace of mind is put at risk from discarding a favored product. But they might adhere to PAO if using expired cosmetics might damage either their image among friends, or their self‐image. The perception of SR or PSR is therefore associated with image consciousness and can cut both ways in terms of its effect, making disposal more likely where the consumer does not want to be perceived (by others or by themselves) as cheap, and making disposal less likely where the product provides a certain cachet associated with being fashionable. Which of these perceived SR and PSR wins out, leading either to the ignoring of or complying with PAO, is likely to depend on a complex set of context‐specific factors.

In general, PHR, PER, and SBCR are more likely to promote adherence to PAO. Consumers discard expired cosmetics when they recognize the potential for skin damage, or when the product no longer functions as intended. SBCR, which is related to the potential enhancement of a consumer–brand relationship through PAO‐related advice, can encourage adherence to the PAO expiry date. However, consumers can rationalize away PHRs as not relevant in a particular case, such as when the use of a cosmetic is minimal, when it is used on less sensitive areas of the face or when it is used on top of another product, all of which are perceived as reducing its potential to cause harm. These inaccurate rationalizations can reduce the impulse to dispose from PHR.

The PAO expiry date may also be ignored when risks interact―for example, when perceived FR or PSR is weighed against PER. The disposal decision can therefore be complex and can vary between individuals depending on the balance between their subjectively perceived and interacting risks. Evidence was presented here that suggests it can also vary by product type (e.g., texture, eye cosmetics or not), by culture and by age, albeit in relatively minor ways. Various heuristics seem to be applied when considering the risk from the use of an expired product, such as whether it is a powder, or, as we have noted above, whether it is used on a certain part of the face or on top of other products. Such heuristics appear to be quite common.

### 4.1 Issues for suppliers

While legislation relevant to PAO varies globally (Halla et al., 2018), responsibility for the safety of cosmetics rests primarily with the provider, which is normally the manufacturer, who will in turn define the PAO. The length of a PAO may be relevant to the marketing of the cosmetic and consequently, the supplier may vary the level of preservative to achieve a longer PAO. Apart from the implications this may have for PHR to the user from product deterioration and sensitivity to preservatives, three other risk factors emerged as relevant to the manufacturer: TR, ENR, and SBCR. While TR was the least significant risk factor in the context of this research, it had relevance to providing information to consumers and to the lack of available PAO information for online sales. Considering the trend of increasing online sales (Gerstell et al., 2020), it is difficult to understand why companies include lists of ingredients for individual products on their websites but do not provide PAO information. This is certainly an area where regulators and policymakers could step in to reduce the risks associated with expired cosmetics. There is no justification in online consumers being disadvantaged in this way.

### 4.2 Two new risk factors

Authors differ in how many dimensions of perceived risk there are (Dowling, 1986; Mitchell, 1992) and there is no theoretical limit on how many there might be. This research adds two: SBCR and ENR. It makes economic sense for manufacturers to build positive associations with their brand and avoid damaging it by creating disaffection among consumers. Clearly, SBCR can be affected by poor communication of a PAO expiry date. There is an opportunity for cosmetics companies to help consumers manage their cosmetics’ expiry (quote SBCR3). This is not merely an economic‐ or marketing‐related concern relevant only to the producer because it would at the same time reduce the risk to the consumer from using expired cosmetics. Similar opportunities were identified in recycling through the quotes provided to substantiate ENR (ENR2 and ENR3). Cosmetics companies are often among those accused of greenwashing (Riccolo, 2021). Recognizing the apprehensions of consumers about the disposal and recycling of unused products as well as (used) packaging should be a concern. There is much to be gained by cosmetics companies from promoting environmentally friendly credentials, but to be effective these might require independent verification by trusted bodies.

### 4.3 Policy implications

As this and prior research (Giacomel et al., 2013; Özdemir et al., 2019; Peony, 2024) shows, the existing policy on consumer protection is not working when it comes to cosmetics. It is therefore essential to provide consumers with more effective guidance on cosmetics expiration.

While all consumers in this study accepted the idea of cosmetics deteriorating after their PAO, leading to lower functionality and product performance and potential risk to health, some qualified or rationalized their positions (e.g., PHR3). The lack of detailed awareness (Cho et al., 2017) and a reluctance to fully accept the consequences of ignoring PAO (Bashir & Lambert, 2020) imply a substantial information campaign is needed. Rothman and Kiviniemi (1999) suggested providing people with broader information than just statistics to help them recognize a potential health problem. The present research also suggests a broad approach is needed, which would include making consumers cognizant of the risk to a healthy and attractive appearance that the use of cosmetics is designed to achieve. Our research implies that cosmetics users would be susceptible to messaging that focuses not only on health risk but also on other risks, particularly SR and PSR. For example, the SR associated with image consciousness could be harnessed to increase compliance with the PAO expiry date. A credible spokesperson could also challenge the logic of seeing FR as an excuse for not disposing of an out‐of‐date product by emphasizing that the risk to one's health has a greater value. There is little to be gained by the cosmetics industry in sponsoring such initiatives (unless they believe that this might lead to increased consumption) raising the issue of how they might be funded.

### 4.4 Eye cosmetics

A specific area for focus is the application of eye cosmetics (e.g., mascara, eye shadow), where health‐related risks (e.g., eye infection) are more obvious from using expired cosmetics. Adverse reactions from expired makeup are mainly related to eye cosmetics (Giacomel et al., 2013). Prior work suggests eye shadow showed the highest degree of microbial contamination among other types of expired cosmetics (Skowron et al., 2017). Mascara typically has the shortest PAO, with FDA (2022) suggesting replacement 3 months after purchase. Ng et al. (2016) concluded that eye care practitioners need to be more aware of the risks posed by eye cosmetics use and remind patients to regularly replace eye cosmetics to avoid contamination. One recommendation could be to publicize adverse events associated with expired eye cosmetic products (Sullivan et al., 2023).

### 4.5 Technical solutions

The main practical problem facing the consumer is remembering when a cosmetic was first used because, if they do not remember, the PAO has no useful meaning. Several solutions have been proposed. Scanning each product into a database is one possibility. The supplier (the retailer or manufacturer) could maintain a database of when a product was purchased and could send reminders to customers when the product reaches its PAO, assuming it was used shortly after purchase. Crosby's (2012) study demonstrates a system that displays expiration months on containers, allowing consumers to set and track expiration dates. For specialized formulas, packaging in individual ampoules can extend shelf life (Cosgrove, 2015). Improving the prominence of PAO information could be useful (Malinowska, 2020), though space constraints on packaging may limit this. Ultimately, the most comprehensive approach currently available is for consumers to record the first day of use along with the product's PAO and refer to this information regularly.

### 4.6 Robustness checks

One reason for using age as a criterion for selecting consumer respondents was our expectation that age would be implicated in disposal decision‐making. In Section 3, we provide several quotations where a consumer's age appeared relevant (FR3, PHR1, PHR4, PSR1, SR2, and SR3). In some, they imply older women would be more likely to adhere to a PAO (PHR1, PHR4, and PSR1) in others the opposite (FR3, SR2, and SR3). This suggests that while the influence of individual risk factors on specific disposal decisions can vary by age, it may not do so systematically.

We interviewed in two countries. Generally, there was little difference between the content of the two sets of interviews other than that ENR emerged from those conducted in the United Kingdom and not China, and we cite prior work to support the idea that the importance of this dimension could be to some degree country‐specific. We used both individual and group interviews, a mixed‐methods approach that might appear controversial, although it has become generally accepted in qualitative research (Morse, 2009; Patton, 2002). Here, it proved useful when respondents knew each other well and insights could emerge that might not have otherwise (e.g., SBCR4 and SBCR5 in Section 3.7). Researchers should feel able to use multiple methods when justified by their context (Morse, 2009). We interviewed industry representatives as well as consumers. While we focus on providing quotes from the latter, salespeople were useful in providing overall comments that improved our understanding of the industry and consumer behavior and in providing a different perspective, as evidenced in quotes SR2 and SR3. Similarly, the interviews with two senior managers provided higher‐level insights and helped to validate our findings. As would have been typical of much research at the time due to COVID, we used face‐to‐face as well as online interviews but found no substantive differences in the insights gained from the two, although we would agree with Wakelin et al. (2024) that analysis is made easier with a video record.

## 5 LIMITATIONS AND FUTURE WORK

Our sample was of just 33 respondents in two countries, but we feel able to make policy suggestions as our work identifies why consumers may ignore the PAO expiry date. Further research on the scale of infection from using expired cosmetics would still be useful. Our sample was exclusively female and further work could usefully include male respondents as their attitudes and motivations for using cosmetics differ (Souiden & Diagne, 2009). This research was conducted only in the United Kingdom and China and research in other cultures would be useful, including those where religious beliefs and cultural traditions can influence cosmetics use.

Our initial objective was to understand why cosmetics users can ignore expiry dates and not to justify any particular risk‐perception framework, avoiding the challenge of confirmation bias. We add to theory by noting that risk factors appear to interact, and examples were cited here of where an issue could only be fully scoped by referring to more than one risk factor. Future quantitative work should consider how independent individual risk factors really are when it comes to the use of cosmetics beyond their expiry date. This includes both the new factors identified here: ENR and SBCR, which need further validation. Finally, work is needed to derive and test messaging based on our work that might best influence cosmetics users to be more cognizant of shelf life issues.

## Footnotes

1 CFG (2024) employ Hofstede's (2001) widely used model for comparing cultural differences between countries.
